# Prothrombotic clot properties can predict venous ulcers in patients following deep vein thrombosis: a cohort study

**DOI:** 10.1007/s11239-019-01914-w

**Published:** 2019-08-20

**Authors:** Maciej Wiktor Polak, Jakub Siudut, Krzysztof Plens, Anetta Undas

**Affiliations:** 1 Institute of Cardiology, Jagiellonian University Medical College, 80 Pradnicka St, 31-202 Krakow, Poland; 2 Krakow Centre for Medical Research and Technologies, John Paul II Hospital, Krakow, Poland; 3 KCRI, Krakow, Poland

**Keywords:** Venous ulcer, Post-thrombotic syndrome, Fibrin, Fibrinolysis, Thrombosis

## Abstract

Venous ulcers are the most severe manifestation of post-thrombotic syndrome (PTS). We have previously demonstrated that formation of compact fibrin clots resistant to lysis is observed in patients following deep-vein thrombosis (DVT) who developed PTS. The current study investigated whether unfavourable fibrin clot properties can predict post-thrombotic venous ulcers. In a cohort study on 186 consecutive patients following DVT, we determined plasma fibrin clot characteristics, including clot permeability and lysability, inflammatory markers, thrombin generation, fibrinolysis proteins at 3 months since the index event. Occurrence of PTS and venous ulcers was recorded during follow-up (median, 53; range 24 to 76 months). Fifty-seven DVT patients (30.6%) developed PTS, including 12 subjects (6.45%) with a venous ulcer (4 individuals with recurrent ulcers). Patients who developed ulcers compared with the remainder had at enrolment 13.0% lower clot permeability (K_s_), 17.4% longer clot lysis time (CLT), 13.1% longer lag phase of clot formation, and 5.0% higher maximum absorbance, with no difference in fibrinogen, C-reactive protein, and thrombin generation. The baseline prothrombotic fibrin clot phenotype (K_s_ ≤ 6.5 × 10^−9^ cm^2^ and CLT > 100 min) was associated with a higher risk of ulcers [hazard ratio (HR), 5.37; 95% confidence interval (CI), 1.3–21.5]. A multivariate model adjusted for age, sex, and fibrinogen showed that independent predictors of the ulcer occurrence were body mass index (HR 1.53; 95% CI 1.30–1.86), CLT (HR 1.43; 95% CI 1.04–2.05), and α_2_-antiplasmin (HR 0.95; 95% CI 0.90–0.99). This study suggests that formation of denser fibrin clots with impaired fibrinolysis predisposes to post-thrombotic venous ulcers.

**Electronic supplementary material:**

The online version of this article (10.1007/s11239-019-01914-w) contains supplementary material, which is available to authorized users.

## Highlights

- Post-thrombotic syndrome (PTS) is a common sequela of deep-vein thrombosis (DVT).
- Prothrombotic clot properties, including dense fibrin networks resistant to lysis, have been observed in patients with DVT who developed PTS during follow-up.
- The most severe manifestation of PTS is a venous ulcer. The known risk factors of venous ulcer formation are older age, obesity and the presence of reflux in deep veins of the lower extremity.
- Patients who develop venous ulcers during follow-up tend to display a specific fibrin clot phenotype, including reduced clot permeability and susceptibility to lysis, at 3 months since the index DVT.
- Prolonged clot lysis time (CLT), lower α_2_-antiplasmin and elevated body mass index were identified as independent predictors of the post-thrombotic venous ulcer. It can be speculated that measurement of clot permeability and CLT might be useful in identifying DVT patients at high risk of post-thrombotic ulcers.

## Introduction

A venous ulcer is a debilitating manifestation of severe post-thrombotic syndrome (PTS) following deep-vein thrombosis (DVT). PTS develops in 20% to 50% of DVT patients, and severe PTS, including venous ulcers, develops in 5% to 10% [1]. The prevalence of venous ulcers has been estimated to range between 0.1 and 2% [2, 3]. Management of venous ulcers is a challenge for a patient and healthcare system [4], given their recurrences in 26–69% of patients within 12 months [5].

Venous ulcers tend to form in the gaiter area of the leg, shallow, surrounded by oedematous tissue and dilated veins [6]. The venous leg ulcer has been postulated to arise from a chronic inflammatory injury secondary to sustained venous hypertension [7]. The development of venous ulcers might result from primary venous valvular reflux or be secondary to previous DVT event [4].

The principal risk factors for PTS are anatomically extensive DVT, failure of vein recanalization, recurrent ipsilateral DVT, persistent leg symptoms 1 month after acute DVT, obesity, and older age [1]. Risk factors for ulcers involve age [8], presence of the reflux in the deep veins [9], high body mass index (BMI), and low physical activity [10], as well as potentially male sex, smoking, diabetes, peripheral arterial disease and leg injury [11]. Anticoagulant therapy and use of compression stockings do not decrease the risk of ulcer development [12].

Blood coagulation eventually results in formation of fibrin clots and their characteristics, reflected by its density and lysability, differ significantly among individuals in association with various genetic and environmental factors [13]. It has been shown that clots composed of thin and highly branched fibrin fibres predispose to arterial and venous thromboembolic events and might have a predictive value in terms of recurrent episodes [14–16]. In 2016 we found that the prothrombotic plasma clot phenotype detected about 3 months since acute DVT is associated with PTS during follow-up [17]. More compact fibrin clot phenotype was also associated with higher plasma D-dimer, C-reactive protein (CRP) and thrombin-activatable fibrinolysis inhibitor (TAFI). However, none of those patients had venous ulcers.

We hypothesised that the unfavourable fibrin clot phenotype increases the risk of developing venous ulcers in DVT patients. For this reason, we performed a cohort study among DVT patients with a long-term follow-up to assess association between fibrin properties and venous ulcers.

## Patients and methods

We studied 197 patients with documented first-ever DVT. Patient characteristics were presented in detail previously [17]. They were referred to an outpatient clinic for further diagnostic work-up between October 2008 and June 2010. The diagnosis of DVT was established by a positive finding on colour duplex sonography. The diagnosis of pulmonary embolism was based on the presence of typical symptoms and positive results of high-resolution spiral computed tomography. A venous thromboembolism (VTE) episode was defined as unprovoked if the patient had no history of cancer, surgery requiring general anaesthesia, major trauma, a plaster cast or hospitalization within the last month, or pregnancy or delivery within the last 3 months. Patients were treated with heparin followed by vitamin K antagonist (VKA).

### Laboratory investigations

Blood samples were collected after fasting from individuals after about 3 months of anticoagulant therapy following the DVT diagnosis. Prior to the testing, patients receiving VKA treatment were switched to heparin for 10–14 days. 12 h following the last injection, when the INR was < 1.2, samples were drawn. Detailed methodology was presented previously [17]. To evaluate lipid profiles, complete blood count, glucose, creatinine and INR, routine laboratory techniques were used. Fibrinogen was determined using the Clauss assay. High-sensitivity CRP was measured by immunoturbidimetry (Roche Diagnostics, Mannheim, Germany). Plasma D-dimer was measured with the Innovance D-dimer assay (Siemens, Marburg, Germany). Plasminogen activator inhibitor-1 (PAI-1) antigen (American Diagnostica, Stamford, CT, USA) was measured using an ELISA, while α_2_-antiplasmin (α_2_AP) and plasminogen were measured by chromogenic assays (STA Stachrom antiplasmin and STA Stachrom plasminogen, Diagnostica Stago, Asnières, France). Plasma-activated TAFI was measured by a chromogenic assay using the ACTICHROME^®^ Plasma TAFI Activity Kit (American Diagnostica). We measured activated and inactivated TAFI (TAFIa/ai) antigen in plasma using a commercially available ELISA kit (Imubind TAFIa/ai antigen ELISA; American Diagnostica) and results were expressed as percentage of pooled plasma from healthy volunteers. Plasma FVIII activity was determined using a one-stage clotting assay (Siemens).

To evaluate clot properties and thrombin generation, venous blood samples (vol/vol, 9:1 of 3.2% trisodium citrate) were centrifuged at 2000×*g* for 10 min within 30 min of the draw, and the supernatant was aliquoted and stored at – 80 °C until analysis. All measurements were performed by technicians blinded to the origin of the samples.

Plasma thrombogenic potential was assessed with calibrated automated thrombography (CAT) (Thrombinoscope BV, Maastricht, the Netherlands) in a 96-well plates fluorometer (Ascent Reader, Thermolab Systems OY, Helsinki, Finland) [18]. Fibrin clot permeation was determined by means of a pressure-driven system [19, 20]. Based on the volume of a buffer flowing through a clot, permeability coefficient (K_s_) was calculated. Lysability of clots was determined by measuring clot lysis time (CLT), defined as the time from the midpoint of the clear-to-maximum-turbid transition to the midpoint of the maximum-turbid-to-clear transition, and by measuring the maximum rate of increase in D-dimer levels (D-D_rate_) and maximum D-dimer concentrations (D–D_max_) during clot perfusion with rtPA [19, 21, 22].

To determine turbidity measurements, plasma citrated samples were mixed with human thrombin (Sigma-Aldrich) and CaCl_2_ and absorbance was read at 405 nm as described [23].

### Genotyping

The FV Leiden, prothrombin 20210A, FXIII Val34Leu and α-fibrinogen Thr312Ala polymorphisms were determined as described [19].

### Follow-up

The patients completed a follow-up period of a minimum of 24 months since enrolment. At their last visit they were assessed using a Villalta scale in order to determine the presence and severity of PTS. Patients were diagnosed with PTS if the Villalta score was at least 5. A score of 5–9 points was categorised as mild, 10–14 as moderate, and 15 and more as severe manifestation of the disease [24].

Every 6 months participants were examined at the clinic in terms of the presence of a venous ulcer. In individuals presented with an ulcer additional data on time of its appearance, healing and recurrence were collected. The patients were examined in terms of the recurrence of DVT during follow-up. Use of compression stockings and anticoagulation treatment were also monitored.

### Statistical analysis

The study was powered to have a 80% chance of detecting a 16% difference in CLT using a one-sided *P* value of 0.025, based on the values of clot lysis time in the previous study [17]. In order to demonstrate such a difference, or a greater one, in total 180 subjects were required, including 12 patients with ulceration.

Categorical variables are presented as numbers and percentages. Continuous variables are expressed as mean ± standard deviation (SD) or median and interquartile range (IQR). Normality was assessed by the Shapiro–Wilk test. Equality of variances was assessed using the Levene’s test. Differences between groups were compared using the Student’s or the Welch’s *t* test depending on the equality of variances for normally distributed variables. The Mann–Whitney *U*-test was used for non-normally distributed continuous variables. Categorical variables were compared by the Pearson’s Chi squared test or the Fisher’s exact test if 20% of cells had an expected count of < 5. Univariate and multivariate Cox proportional hazard analysis was performed to identify independent predictors of the outcomes. The multivariate models were fitted using backward stepwise regression. Variables that were associated with the occurrence of the outcomes in univariate models with a significance level of *P* < 0.25 were selected for possible inclusion in the multivariate logistic regression model. The log-rank statistic was used to test the differences in the outcomes between the groups. Two-sided *P*-values < 0.05 were considered statistically significant. All calculations were done with JMP^®^, Version 14.2.0 (SAS Institute Inc., Cary, NC, USA).

## Results

Since eleven patients were lost to follow-up, a total of 186 patients were included in the final analysis. Characteristics of the study population were shown in Table 1.

**Table 1:** Baseline patient characteristics

Variables	Total cohort (n = 186)	Patients with venous ulcer (n = 12)	Patients free of venous ulcer (n = 174)	*P*
Age (years)	45 (33–54.25)	47.5 (39.5–60.75)	45 (32–53.25)	0.147
Male [n (%)]	101 (54.3)	4 (33.3)	97 (55.8)	0.132
Body mass index (kg/m^2^)	26.85 (24.9–29.2)	33.75 (30.6–36.05)	26.65 (24.88–28.93)	< 0.001
Obesity [n (%)]	28 (15.05)	10 (83.3)	18 (10.3)	< 0.001
Active smoking [n (%)]	73 (39.3)	3 (25)	70 (40.2)	0.371
Family history of VTE [n (%)]	28 (15.1)	2 (16.7)	26 (14.9)	1.000
Clinical variables [n (%)]				
Post-Trauma VTE	38 (20.4)	0 (0)	38 (21.8)	0.130
Unprovoked VTE	91 (48.9)	7 (58.3)	84 (48.3)	0.500
DVT alone	57 (30.7)	3 (25)	54 (31)	0.758
PE + DVT	129 (69.3)	9 (75)	120 (69)	
Proximal DVT	138 (74.2)	11 (91.7)	127 (73)	0.192
Anticoagulant therapy	44 (23.7)	10 (83.3)	34 (19.5)	< 0.001
Compression therapy	22 (11.8)	9 (75)	13 (7.5)	< 0.001
Laboratory investigations at enrolment				
INR	0.98 (0.9–1.05)	1.01 (0.97–1.07)	0.98 (0.90–1.05)	0.097
D-dimer (ng/mL)	214 (160–283)	241 (213–265)	211 (158–287)	0.138
Fibrinogen (g/L)	2.98 (2.48–3.87)	3.03 (2.67–4.05)	2.98 (2.45–3.87)	0.544
Creatinine (μmol/L)	71.18 ± 13.51	70.92 ± 11.05	71.20 ± 13.69	0.944
Glucose (mmol/L)	4.9 (4.58–5.3)	5.10 (4.85–5.48)	4.90 (4.51–5.30)	0.174
Triglycerides (mmol/L)	1.16 (0.71–1.72)	1.34 (0.90–1.68)	1.15 (0.71-1.72)	0.754
TC (mmol/L)	5.05 (4.2–5.78)	4.91 (4.43–5.93)	5.07 (4.19–5.78)	0.831
LDL-C (mmol/L)	3.04 ± 0.86	3.09 ± 0.82	3.04 ± 0.87	0.845
HDL-C (mmol/L)	1.40 (1.14–1.69)	1.32 (0.99–1.66)	1.40 (1.14–1.71)	0.326
CRP (mg/L)	1.70 (1.02–2.34)	2.64 (1.16–7.45)	1.61 (1.02–2.31)	0.059
Peak thrombin, nM	233 (198–305)	230 (188–307)	233 (199–305)	0.544
Factor VIII (%)	124 (103–142)	120 (111–143)	124 (102–142)	0.912
PAI-1 (ng/mL)	12.10 (8.79–18.05)	9.64 (8.27–17.65)	12.25 (8.88–18.35)	0.353
TAFI activity (µg/mL)	25.55 (20.20–30.30)	31.41 (26.12–39.67)	25.15 (20.14–29.97)	0.004
TAFI antigen (%)	100 (89–110)	108 (96–116)	100 (89–110)	0.066
Plasminogen (%)	107 (97–120)	106 (91–119)	107 (98–120)	0.392
α_2_-antiplasmin (%)	103 (96–116)	97 (90–103)	104 (97–116)	0.022
Genetic polymorphisms [n (%)]				
Factor V Leiden	24 (12.9)	1 (8.3)	23 (13.2)	1.00
Prothrombin 20210A	7 (3.8)	0 (0)	7 (4)	1.00
Factor XIII Val34Leu	84 (45.2)	6 (50)	78 (44.8)	0.728
α-fibrinogen Thr312Ala	82 (44.1)	8 (66.7)	74 (42.5)	0.103

Data are shown as mean ± SD or a median (interquartile range) or number (percentage)

*VTE* venous thromboembolism, *PTS* post-thrombotic syndrome, *DVT* deep vein thrombosis, *PE* pulmonary embolism, *INR* international normalized ratio, *TC* total cholesterol, *LDL*-*C* low-density lipoprotein cholesterol, *HDL*-*C* high-density lipoprotein cholesterol, *CRP* C-reactive protein, *PAI*-*1* plasminogen activator inhibitor-1, and *TAFI* thrombin-activatable fibrinolysis inhibitor

After a median follow-up of 53 months, PTS was diagnosed in 57 subjects (30.6%), including 21 cases of mild (11.3%), 12 cases of moderate (5.9%) and 24 (12.9%) cases of severe PTS. Venous ulcers occurred in 12 (6.45%) individuals. The presence of an ulcer on the lower extremity persisted for 6 to 17 months (10 months, median). In one case the ulcer remained unhealed until the end of observation period. Four patients (2.15%) suffered from recurrent ulcers. Individuals who developed ulcers were more frequently obese compared to the remainder, with no other demographics and clinical intergroup differences (Table 1). Compression therapy and anticoagulant therapy were reported by most patients in the ulcer group compared with the non-ulcer group (Table 1).

Analysis of haemostatic markers showed that the occurrence of ulcers was associated with lower concentration α_2_-antiplasmin and higher TAFI activity at enrolment (Table 1). There were no intergroup differences in fibrinogen, CRP and thrombin generation. The four genetic polymorphisms tested were similarly distributed in the two groups (Table 1).

As shown in Table 2, at 3 months since the DVT diagnosis, K_s_ was 13.0% lower in the patients who developed venous ulcers during follow-up as compared to the remainder, while CLT was 17.4% longer in this group. Furthermore, we observed 13.1% longer lag phase and 5% greater ΔAbs in the ulcer group compared with the non-ulcer group (Table 2). Both D–D_max_ and D-D_rate_ values showed no intergroup differences. The so-called prothrombotic phenotype of the fibrin clot, defined as the lower quartile of K_s_ (≤ 6.5 × 10^−9^ cm^2^) and the top quartile of CLT (> 100 min), was associated with a 5.37-fold [95% confidence interval (CI) 1.3–21.5] higher risk of venous ulcers in patients with DVT during follow-up.

**Table 2:** Comparison of fibrin clot properties

Variable	Total cohort (n = 186)	Patients with venous ulcer (n = 12)	Patients free of venous ulcer (n = 174)	*P*
K_s_, 10^−9^ cm^2^	7.45 ± 1.27	6.53 ± 0.45	7.51 ± 1.28	< 0.001
CLT (min)	88 (72–100)	101 (88–114)	86 (72–99)	0.007
Lag phase (s)	41 (37–46)	36.5 (35–39.75)	42 (38–46)	0.003
ΔAbs (405 nm)	0.81 (0.75–0.86)	0.84 (0.81–0.9)	0.8 (0.74–0.86)	0.03
D-D_max_ (mg/L)	3.99 (3.60–4.39)	3.68 (3.59–3.99)	4.07 (3.6–4.39)	0.132
D-D_rate_ (mg/L/min)	0.072 ± 0.008	0.072 ± 0.009	0.072 ± 0.008	0.68

Values are given as mean ± SD or a median (interquartile range)

K_s_ indicates permeability coefficient; *CLT* clot lysis time, *ΔAbs* maximum absorbance, *D*-*D*_*max*_ maximum D-dimer levels in the lysis assay, and *D*-*D*_*rate*_ maximum rate of increase in D-dimer levels in the lysis assay

Univariate analysis showed that BMI, recurrent VTE, using of compression prior to ulcer, anticoagulation, CRP, ΔAbs, CLT and TAFI activity were positively associated with the risk of a venous leg ulcer, and shorter lag phase, K_s_, and α_2_-antiplasmin increased the risk of venous ulcer occurrence (data not shown). A multivariate model showed that CLT, BMI and α_2_-antiplasmin are predictors of the ulcer occurrence (Supplementary Table S1).

Patients who developed PTS presented in Supplementary Table S2 were characterised by a 10% lower K_s_, 14% longer CLT, 9% shorter lag phase, and 4% higher ΔAbs, all typical of the prothrombotic fibrin clot phenotype (Supplementary Table S3).

## Discussion

To our knowledge, this study is the first to show association of the plasma fibrin clot phenotype and venous ulcers among DVT patients. We found that denser fibrin clot formation and reduced susceptibility to lysis not only predispose to PTS, which is in line with our previous study with a shorter follow-up of 12 months [17], but also significantly increase the risk of a post-thrombotic venous ulcer. Our findings suggest that unfavourable fibrin clot properties may have a predictive value in terms of venous ulcers.

Analysis of several proteins involved in fibrinolysis in the context of post-thrombotic leg ulcers provided additional interesting observations. We noted that DVT patients with venous ulcers had higher baseline TAFI activity accompanied by lower α_2_-antiplasmin compared with the remaining subjects, without any differences in PAI-1 or plasminogen. The observation on TAFI is consistent with our previous study, where hypofibrinolysis was associated with higher TAFI activity in PTS patients [17]. The current finding supports involvement of elevated TAFI activity in PTS. Surprisingly, in individuals in the ulcer group we observed lower α_2_-antiplasmin levels, but all within the reference range, without any difference in this parameter related to the presence or absence of PTS. This key fibrinolysis inhibitor [25] accounts for about 90% of plasmin inhibition in vivo [26]. Since the resistance of thrombi to plasmin digestion depends primarily on the amount of α_2_AP incorporated within fibrin, lower antifibrinolytic antiplasmin, though linearly not correlated to CLT, might suggest that increased, not impaired, fibrinolysis enhances the post-thrombotic ulcer formation. However, we found that prolonged CLT is associated with this complication of DVT, therefore it is unlikely that antiplasmin-mediated impact on global efficiency of fibrinolysis explains the protective effect of lower α_2_AP on ulcer formation. Lower antiplasmin could indicate changes in a physiological ratio of two forms of this protein [27]. Antiplasmin-cleaving enzyme (APCE) cleaves Met-α_2_AP to Asn-α_2_AP, which is more efficiently incorporated into fibrin and makes it resistant to plasmin digestion. Another potential factor affecting the antiplasmin could be α_2_AP posttranslational modifications such as oxidation that may modulate the protein’s function [28]. Mechanisms underlying the role of antiplasmin in the context of post-thrombotic ulcers require further research. We cannot exclude that modulation of antiplasmin activity might prevent venous ulcer formation.

Looking at several demographic and clinical factors and their associations with post-thrombotic ulcers, we identified obesity as the strongest predisposing factor, which agrees with several papers [9, 10]. Galanaud et al. found that the male sex increases the risk of ulcers after VTE at 1 year only, whereas after 2 or 3 years of follow-up this association was no longer significant [12]. We did not observe a higher proportion of males in the ulcer group during a few years since DVT, which is similar to that study. Since diabetic patients had been excluded from our study due to prothrombotic clot alterations observed commonly in diabetes [17, 29], the impact of this disease on post-thrombotic ulcers was not evaluated in the present study. Anticoagulant therapy and the use of compression stockings did not reduce the risk of venous ulceration [12], which agrees with our findings.

We found higher CRP levels of borderline statistical significance in the ulcer group, while fibrinogen concentrations were similar. Previous studies demonstrated that elevated plasma levels of inflammatory biomarkers, including CRP, were associated with PTS [30, 31]. This study supports the view that elevated CRP is not related to the risk of leg ulceration. Nonetheless, increased CRP levels in patients with ulcers might indicate the presence of infection in affected tissues [32]. On the other hand, we confirmed that the presence of PTS has been linked to systemic inflammation. We did not observe any impact of heightened thrombin generation at baseline on development of venous ulcers in follow-up, therefore elevated thrombin, the well-known factor leading to more compact clot network formation [33], does not contribute to unfavourable fibrin clot properties in this patient group.

This study has several limitations. Although the study was adequately powered, the number of patients with venous ulcers was limited, however the prevalence of post-thrombotic ulcers is low as shown in other studies [34, 35]. Our study was designed to assess fibrin clots in relation to PTS in patients below 70 years, free of severe thrombophilia, cancer or diabetes to avoid factors adversely affecting clot phenotype [22, 36, 37]. Our results cannot be easily extrapolated to those patient subsets. Molecular mechanisms underlying elevated risk of post-thrombotic ulcers in the presence of the prothrombotic clot phenotype were beyond the scope of the current study. It has been shown that decreased K_s_ not only correlates with thromboembolic events, but also is associated with an elevated bleeding risk and, consequently, with disturbances in healing of mild skin injuries [38]. This observation may explain at least in part the persistence of venous ulcers in individuals characterised by unfavourable fibrin clot phenotype.

The current study demonstrates that patients who develop venous ulcers following DVT produce dense and lysis-resistant fibrin clots. It might be speculated that measurement of clot permeability and CLT might be useful in identifying DVT patients at high risk of post-thrombotic ulcers. How to reduce this risk should be a subject of further investigation.

## Electronic supplementary material

Below is the link to the electronic supplementary material.

Supplementary material 1 (DOCX 13 kb)

Supplementary material 2 (DOCX 19 kb)

Supplementary material 3 (DOCX 13 kb)
