# Supplementary material for: Prothrombotic clot properties can predict venous ulcers in patients following deep vein thrombosis: a cohort study
Source: J Thromb Thrombolysis. 2019 Aug 20;48(4):603–9. doi: 10.1007/s11239-019-01914-w (PMC6800839; doi:10.1007/s11239-019-01914-w)
Supplement: Supplementary file 1 — Supplementary material 1 (DOCX 13 kb) [file 11239_2019_1914_MOESM1_ESM.docx]

**Supplementary Table S1.** The Cox regressions multivariate model of risk predictors of venous ulcers

|  |  | **Multivariate*** |  |
| --- | --- | --- | --- |
| **Variable** | **HR per** | **HR (95% CI)** | ***P*** |
| BMI | 1 kg/m^2^ | 1.53 (1.30-1.86) | <0.001 |
| CLT | 10 min | 1.43 (1.04-2.05) | 0.029 |
| α_2_-antiplasmin | 1 % | 0.95 (0.90-0.99) | 0.036 |

* Adjusted for age, sex and fibrinogen.

HR, hazard ratio; CI, confidence interval; BMI, body mass index; CLT, clot lysis time.
