# Supplementary material for: Prothrombotic clot properties can predict venous ulcers in patients following deep vein thrombosis: a cohort study
Source: J Thromb Thrombolysis. 2019 Aug 20;48(4):603–9. doi: 10.1007/s11239-019-01914-w (PMC6800839; doi:10.1007/s11239-019-01914-w)
Supplement: Supplementary file 2 — Supplementary material 2 (DOCX 19 kb) [file 11239_2019_1914_MOESM2_ESM.docx]

**Supplementary Table S2.** Characteristics of patients with and without PTS

| **Variables** | **Total cohort (n = 186)** | **PTS (n = 57)** | **Non-PTS**  **(n = 129)** | ***P*** |
| --- | --- | --- | --- | --- |
| Age, years | 45 (33-54) | 49 (40-58) | 43 (32-52) | 0.007 |
| Male, n (%) | 101 (54.3) | 30 (52.6) | 71 (55.0) | 0.76 |
| Body mass index, kg/m^2^ | 26.3 (24.6-29.0) | 27.3 (25.5-30.1) | 25.9 (24.3-28.3) | 0.006 |
| Active smoking, n (%) | 73 (39.3) | 18 (31.6) | 55 (42.6) | 0.15 |
| Family history of VTE, n (%) | 28 (15.1) | 8 (14.0) | 20 (15.5) | 0.80 |
| **Clinical variables, n (%)** | | | | |
| Post-Trauma VTE | 38 (20.4) | 7 (12.3) | 31 (24.0) | 0.07 |
| Unprovoked VTE | 91 (48.9) | 37 (64.9) | 54 (41.9) | 0.004 |
| DVT alone | 129 (69.4) | 43 (75.4) | 86 (66.7) | 0.23 |
| PE+DVT | 57 (30.6) | 14 (24.6) | 43 (33.3) |  |
| Proximal DVT | 138 (74.2) | 48 (84.2) | 90 (69.8) | 0.004 |
| Anticoagulant therapy | 44 (23.7) | 27 (47.4) | 17 (13.2) | <0.001 |
| Compression therapy | 22 (11.8) | 22 (38.6) | 0 (0) | <0.001 |
| **Laboratory investigations at enrolment** | | | | |
| INR | 0.98 (0.90-1.05) | 1.00 (0.92-1.05) | 0.98 (0.90-1.05) | 0.46 |
| D-dimer, ng/mL | 213 (160-283) | 230 (170-281) | 211 (157-285) | 0.36 |
| Fibrinogen, g/L | 2.98 (2.48-3.87) | 2.93 (2.51-3.94) | 3.03 (2.45-3.84) | 0.87 |
| Creatinine, μmol/L | 71.18±13.51 | 71.91±14.96 | 70.86±12.87 | 0.63 |
| Glucose, mmol/L | 4.90 (4.58-5.30) | 5.00 (4.79-5.50) | 4.80 (4.50-5.20) | 0.002 |
| Triglycerides, mmol/L | 1.16 (0.71-1.72) | 1.26 (0.84-2.01) | 1.06 (0.70-1.54) | 0.023 |
| TC, mmol/L | 5.07±1.07 | 5.08±0.94 | 5.06±1.12 | 0.66 |
| LDL-C, mmol/L | 3.04±0.86 | 3.03±0.75 | 3.05±0.91 | 0.91 |
| HDL-C, mmol/L | 1.40 (1.14-1.69) | 1.40 (1.13-1.69) | 1.40 (1.14-1.71) | 0.92 |
| CRP, mg/L | 1.70 (1.02-2.34) | 1.99 (1.24-3.00) | 1.48 (1.01-2.24) | 0.03 |
| Peak thrombin, nM | 233 (198-305) | 224 (192-293) | 235 (200-306) | 0.30 |
| Factor VIII, % | 124 (103-142) | 121 (103-143) | 124 (102-143) | 0.53 |
| PAI-1, ng/mL | 12.10 (8.79-18.05) | 11.20 (8.47-17.79) | 12.30 (8.91-18.7) | 0.37 |
| TAFI activity, µg/mL | 25.55 (20.20-30.30) | 29.91 (25.75-34.93) | 22.80 (19.80-28.34) | <0.001 |
| TAFI antigen, % | 100 (89-110) | 103 (95-114) | 99 (88-107) | 0.002 |
| Plasminogen, % | 107 (97-120) | 103 (98-118) | 108 (97-120) | 0.32 |
| α_2_-antiplasmin, % | 103 (96-116) | 102 (93-113) | 104 (98-116) | 0.28 |
| **Genetic polymorphisms, n(%)** | | | | |
| Factor V Leiden | 24 (12.9) | 9 (15.8) | 15 (11.6) | 0.44 |
| Prothrombin 20210A | 7 (3.8) | 1 (1.8) | 6 (4.7) | 0.68 |
| Factor XIII Val34Leu | 84 (45.2) | 29 (50.9) | 55 (42.6) | 0.30 |
| α-fibrinogen Thr312Ala | 82 (44.1) | 27 (47.4) | 55 (42.6) | 0.55 |

Data are shown as mean ± SD or a median (interquartile range) or number (percentage).

VTE, venous thromboembolism; PTS, post-thrombotic syndrome; DVT, deep vein thrombosis; PE, pulmonary embolism; INR, international normalized ratio; TC, total cholesterol; LDL-C, low-density lipoprotein cholesterol; HDL-C, high-density lipoprotein cholesterol; CRP, C-reactive protein; PAI-1, plasminogen activator inhibitor-1; and TAFI, thrombin-activatable fibrinolysis inhibitor.
