# Supplementary material for: Prothrombotic clot properties can predict venous ulcers in patients following deep vein thrombosis: a cohort study
Source: J Thromb Thrombolysis. 2019 Aug 20;48(4):603–9. doi: 10.1007/s11239-019-01914-w (PMC6800839; doi:10.1007/s11239-019-01914-w)
Supplement: Supplementary file 3 — Supplementary material 3 (DOCX 13 kb) [file 11239_2019_1914_MOESM3_ESM.docx]

**Supplementary Table S3.** Fibrin clot properties in patients with and without PTS

| **Variable** | **Total cohort (n = 186)** | **PTS (n = 57)** | **Non-PTS**  **(n = 129)** | ***P**** |
| --- | --- | --- | --- | --- |
| K_s_, 10^−9^ cm^2^ | 7.45±1.27 | 6.93±1.12 | 7.68±1.26 | <0.001 |
| CLT, min | 88 (72-100) | 96 (84-107) | 84 (70-99) | <0.001 |
| Lag phase, s | 41 (37-46) | 39 (36-43) | 43 (38-47) | <0.001 |
| ΔAbs (405nm) | 0.81 (0.75-0.86) | 0.83 (0.80-0.88) | 0.80 (0.74-0.86) | 0.001 |
| D-D_max_, mg/L | 3.99 (3.60-4.39) | 3.80 (3.62-4.33) | 4.09 (3.59-4.39) | 0.62 |
| D-D_rate_, mg/L/min | 0.072 (0.069-0.078) | 0.070 (0.066-0.075) | 0.070 (0.068-0.078) | 0.081 |

Values are given as mean ± SD or a median (interquartile range).

K_s_ indicates permeability coefficient; CLT, clot lysis time; ΔAbs – maximum absorbance; D-D_max_, maximum D-dimer levels in the lysis assay; and D-D_rate_, maximum rate of increase in D-dimer levels in the lysis assay.

*Adjusted for age, BMI and fibrinogen level
